# Crystal structures of deuterated sodium molybdate dihydrate and sodium tungstate dihydrate from time-of-flight neutron powder diffraction

**DOI:** 10.1107/S2056989015011354

**Published:** 2015-06-17

**Authors:** A. Dominic Fortes

**Affiliations:** a ISIS Facility, Rutherford Appleton Laboratory, Harwell Science and Innovation Campus, Didcot, Oxfordshire OX11 0QX, England; b Department of Earth Sciences, University College London, Gower Street, London WC1E 6BT, England; c Department of Earth and Planetary Sciences, Birkbeck, University of London, Malet Street, London WC1E 7HX, England

**Keywords:** neutron powder diffraction, sodium molybdate dihydrate, sodium tungstate dihydrate

## Abstract

Time-of-flight neutron powder diffraction data have been measured from ∼90 mol% deuterated isotopologues of Na_2_MoO_4_·2H_2_O and Na_2_WO_4_·2H_2_O at 295 K to a resolution of sin (θ)/λ = 0.77 Å^−1^. The use of neutrons has allowed refinement of structural parameters with a precision that varies by a factor of two from the heaviest to the lightest atoms; this contrasts with the X-ray based refinements where precision may be > 20× poorer for O atoms in the presence of atoms such as Mo and W. The accuracy and precision of inter­atomic distances and angles are in excellent agreement with recent X-ray single-crystal structure refinements whilst also completing our view of the hydrogen-bond geometry to the same degree of statistical certainty. The two structures are isotypic, space-group *Pbca*, with all atoms occupying general positions, being comprised of edge- and corner-sharing NaO_5_ and NaO_6_ polyhedra that form layers parallel with (010) inter­leaved with planes of *X*O_4_ (*X* = Mo, W) tetra­hedra that are linked by chains of water mol­ecules along [100] and [001]. The complete structure is identical with the previously described molybdate [Capitelli *et al.* (2006 ▸). *Asian J. Chem*. **18**, 2856–2860] but shows that the purported three-centred inter­action involving one of the water mol­ecules in the tungstate [Farrugia (2007 ▸). *Acta Cryst.* E**63**, i142] is in fact an ordinary two-centred ‘linear’ hydrogen bond.

## Abstract 2

High-precision structural parameters for Na_2_MoO_4_·2D_2_O and Na_2_WO_4_·2D_2_O are reported based on refinement of high-resolution time-of-flight neutron powder diffraction data. Complementary Raman spectra are also provided.

## Chemical context   

Na_2_MoO_4_ and Na_2_WO_4_ are unusual amongst the alkali metal mono-molybdates and mono-tungstates in being highly soluble in water *and* forming polyhydrated crystals. Additionally, sodium apparently plays a significant role in the solvation of other alkali metal ions to form a range of double molybdate and tungstate hydrates (Klevtsova *et al.*, 1990 ▸; Klevtsov *et al.*, 1997 ▸; Mirzoev *et al.*, 2010 ▸), for example, Na_3_K(MoO_4_)_2_·9H_2_O. Both dihydrate and deca­hydrate varieties of the two title compounds are known, their solubilities as a function of temperature being well characterised (Funk, 1900 ▸; Zhilova *et al.*, 2008 ▸). The structures of the deca­hydrates have not yet been reported, although I have established that they are not isotypic with the sodium sulfate analogue, Na_2_SO_4_·10H_2_O, as had hitherto been thought.

The dihydrates have been the subject of extensive crystallographic studies, from descriptions of their density, habit and measurements of inter­facial angles (Svanberg & Struve, 1848 ▸; Zenker, 1853 ▸; Rammelsberg, 1855 ▸; Marignac, 1863 ▸; Delafontaine, 1865 ▸; Ullik, 1867 ▸; Clarke, 1877 ▸; Zambonini, 1923 ▸), through to determination of absolute unit-cell parameters (Pistorius & Sharp, 1961 ▸), and subsequent solution and refinement of their structures (Mitra & Verma, 1969 ▸; Okada *et al.*, 1974 ▸; Matsumoto *et al.*, 1975 ▸; Atovmyan & D’yachenko, 1969 ▸; Capitelli *et al.*, 2006 ▸; Farrugia, 2007 ▸). However, the presence of heavy atoms in these materials makes it impossible to achieve a uniform precision on all structural parameters using X-rays, and even with single-crystal methods that purport to identify hydrogen positions there may be significant inaccuracies. Such problems are minimised using a neutron radiation probe since the coherent neutron scattering lengths of the constituent elements differ by less than a factor of two, being 6.715 fm for Mo, 4.86 fm for W, 3.63 fm for Na, 5.803 fm for O, and 6.67 fm for ^2^D (Sears, 1992 ▸). Thus one can locate accurately all of the light atoms and obtain a uniform level of precision on their coordinates and displacement parameters. Since the incoherent neutron scattering cross section of ^1^H is large (80.3 barns) it is usual to prepare perdeuterated specimens whenever possible (the incoherent cross section of ^2^D being only 2.1 barns) as this optimises the coherent Bragg scattering signal above the background, reducing the counting times required for a high-precision structure refinement from many days to a matter of hours on the instrument used for these measurements. These data were therefore measured using Na_2_MoO_4_·2D_2_O and Na_2_WO_4_·2D_2_O samples.

The occurrence of polyhydrated forms of both Na_2_MoO_4_ and Na_2_WO_4_ suggests that both would be excellent candidates for the formation of hydrogen-bonded complexes with water-soluble organics, such as amino acids, producing metal-organic crystals with potentially useful optical properties (*cf*., glycine lithium molybdate; Fleck *et al.*, 2006 ▸). High-pressure polymorphs of Na_2_MoO_4_·2H_2_O and Na_2_WO_4_·2H_2_O are indicated from Raman scattering studies (Luz-Lima *et al.*, 2010 ▸; Saraiva *et al.*, 2013 ▸). Characterising the structures and properties of the title compounds provides an essential foundation on which to build future studies of the high-pressure phases, of the as-yet incomplete deca­hydrate structures and any related organic-bearing hydrates.

## Structural commentary   

**Figure 1:**
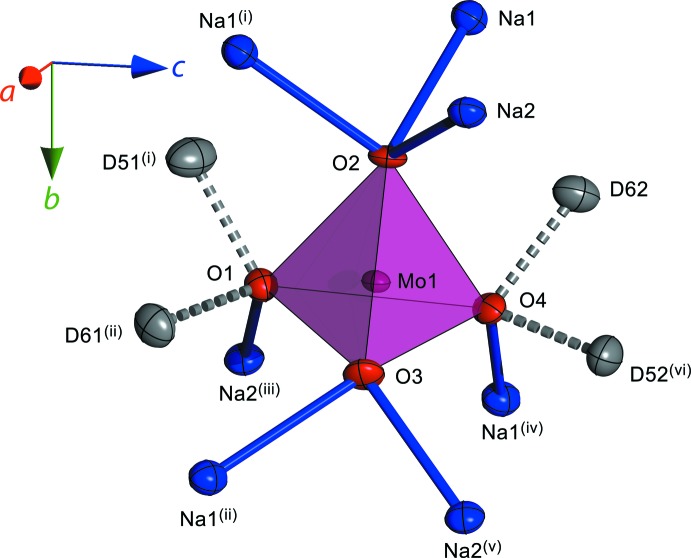
First and second coordination shell of Mo^6+^/W^6+^ in the title compounds, revealing differences in the environment of each apical O^2−^ that are responsible for the variations in Mo–O and W–O bond lengths. Anisotropic displacement ellipsoids are drawn at the 50% probability level. [Symmetry codes: (i) 1 − *x*, 1 − *y*, 1 − *z*; (ii) 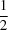 + *x*, 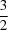 − *y*, 1 − *z*; (iii) −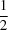 + *x*, 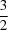 − *y*, 1 − *z*; (iv) 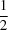 − *x*, 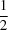 + *y*, *z*; (v) 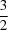 − *x*, 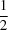 + *y*, *z*; (vi) 1 − *x*, 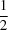 + *y*, 1.5 − *z*.]

Na_2_MoO_4_·2H_2_O and Na_2_WO_4_·2H_2_O are isotypic, crystallizing in the ortho­rhom­bic space group *Pbca*; all atoms occupy general positions (Wyckoff sites 8*c*). Note that the atom labelling scheme and space-group setting used here follows Farrugia (2007 ▸); consequently there are some differences with respect to other literature sources, although equivalent contacts are referred to in Table 1 ▸ and Table 2 ▸. The *X* ^6+^ ions (*X* = Mo, W) are tetra­hedrally coordinated by O^2−^, the Mo—O and W—O bond lengths varying slightly according to the type of coordination adopted by a particular apex: O1 and O4 are each coordinated to Na^+^ and each also accepts two hydrogen bonds; O2 is coordinated to three Na^+^ ions and O3 is coordinated to two Na^+^ ions (Fig. 1 ▸). In both title compounds, *X*–O1 and *X*–O4 are the longest contacts and *X*–O3 is the shortest contact in the tetra­hedral oxyanion. The mean Mo—O and W—O bond lengths are in good agreement with those found in the anhydrous crystals (Fortes, 2015 ▸). Furthermore, each of the absolute Mo—O bond lengths are identical (within error) to those found by Capitelli *et al.* (2006 ▸); the agreement in W—O bond lengths with Farrugia (2007 ▸) is marginally poorer.

**Figure 2:**
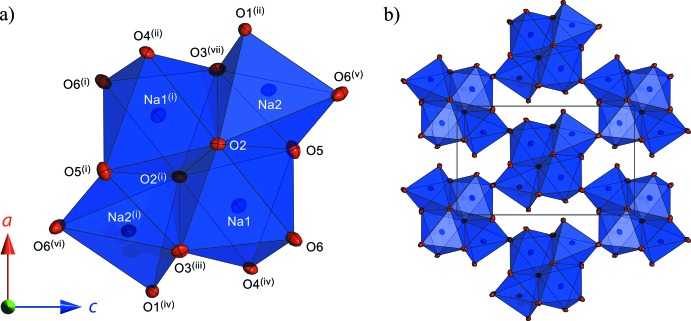
(*a*) Arrangement of NaO_*x*_ polyhedra into edge-sharing clusters comprised of two Na1O_6_ octa­hedra and two Na2O_5_ square pyramids; (*b*) Arrangement of the clusters shown in (*a*) by corner sharing to form ‘slabs’ parallel (010). Ellipsoids are drawn at the 50% probability level. [Symmetry codes: (i) 1 − *x*, 1 − *y*, 1 − *z*; (ii) 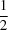 + *x*, 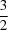 − *y*, 1 − *z*; (iii) −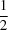 + *x*, 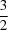 − *y*, 1 − *z*; (iv) 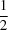 − *x*, −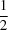 + *y*, *z*; (v) 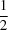 + *x*, *y*, 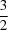 − *z*; (vi) 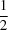 − *x*, 1 − *y*, 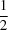 + *z*; (vii) 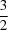 − *x*, −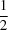 + *y*, *z*.]

The Na^+^ ions occupy two inequivalent sites: in one, Na^+^ is six-fold coordinated by two water mol­ecules and four *X*O_4_^2−^ oxygen atoms, yielding an octa­hedral arrangement; in the second, Na^+^ is five-fold coordinated by two water mol­ecules and three *X*O_4_^2−^ oxygen atoms, yielding a square-pyramidal arrangement. These two polyhedra share a common edge (O2–O5) and are connected, moreover, with their inversion-centre-related neighbours along three other shared edges to form a cluster (Fig. 2 ▸ *a*). The clusters corner-share *via* O6 to create a ‘slab’ parallel to (010) (Fig. 2 ▸ *b*). The mean Na—O bond lengths are statistically identical in Na_2_MoO_4_·2D_2_O and Na_2_WO_4_·2D_2_O being ∼1.6% longer in the NaO_6_ octa­hedra and ∼2.3% shorter in the NaO_5_ polyhedra than Na—O bonds in the anhydrous crystals (Fortes, 2015 ▸). The agreement in Na—O bond lengths with the X-ray single crystal studies of Capitelli *et al.* (2006 ▸) and Farrugia (2007 ▸) is very good. Overall, the agreement in bond lengths and angles for the two independently refined data sets is excellent (Tables 1 ▸ and 2 ▸).

**Figure 3:**
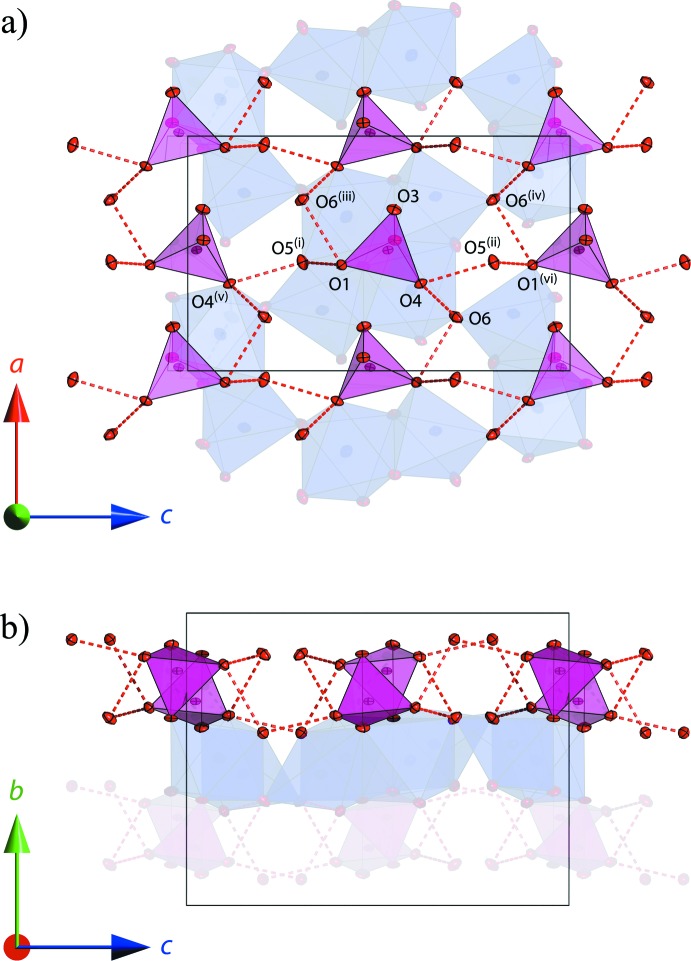
(*a*) View down the *b* axis of the network of water-linked tetra­hedral oxyanions; chains linked by O5 extend along [001] whereas crosslinkages through O6 are staggered along [100]. (*b*) View of the same structure along the *c* axis. Ellipsoids are drawn at the 50% probability level. [Symmetry codes: (i) 1 − *x*, 1 − *y*, 1 − *z*; (ii) 1 − *x*, 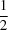 + *y*, 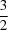 − *z*; (iii) 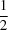 + *x*, 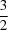 − *y*, 1 − *z*; (iv) 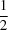 + *x*, *y*, 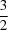 − *z*; (v) *x*, 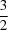 − *y*, −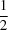 + *z*; (vi) *x*, 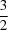 − *y*, 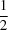 + *z*.]

Although it is more usual to find Na^+^ in octa­hedral coordination, there are abundant examples of Na^+^ in five-fold coordination, including instances where the NaO_5_ polyhedron adopts a square-pyramidal arrangement (Beurskens & Jeffrey, 1961 ▸; Císařová; *et al.*, 2001 ▸; Sharma *et al.*, 2005 ▸; Smith & Wermuth, 2014 ▸; Aksenov *et al.*, 2014 ▸) or the alternative trigonal-bipyramidal arrangement (Mereiter, 2013 ▸; Smith, 2013 ▸). A similar combination of NaO_6_ and NaO_5_ polyhedra to that found in the title compounds occurs in the closely-related hydrates Na_2_CrO_4_·1.5H_2_O and Na_2_SeO_4_·1.5H_2_O (Kahlenberg, 2012 ▸; Weil & Bonneau, 2014 ▸). The two water mol­ecules form hydrogen-bonded chains between the O1 and O4 atoms of the tetra­hedral oxyanions; O5-related chains extend along [001] and O6-related chains crosslink them in a staggered fashion along [100]. Fig. 3 ▸(*a*) and 3(*b*) depict the spatial relationship between this ‘net’ of water linked tetra­hedra and the adjacent ‘slab’ of corner-linked Na—O polyhedral clusters. The layers shown in Fig. 3 ▸(*b*) alternate to create the three-dimensional structure and are no doubt responsible for the macro-scale platy habit of the crystals.

There are no significant differences in the hydrogen bond geometries of the molybdate or tungstate crystals. The most recent X-ray single-crystal diffraction study of Na_2_WO_4_·2H_2_O (Farrugia, 2007 ▸) implied that one of the water mol­ecules (O5) was involved in a weaker three-centred inter­action, although a similarly recent measurement of Na_2_MoO_4_·2H_2_O (Capitelli *et al.*, 2006 ▸) identified a ‘normal’ linear two-centred inter­action for this bond. This work, using neutrons, has been able to accurately and precisely characterise the hydrogen bond geometry, showing that the latter is true for both structures; there is no bifurcated bond and all hydrogen-bonded inter­actions are of the linear two-centred variety. Presumably the error in Farrugia’s analysis arose due to the substantial absorption correction required (μ = 18.7 mm^−1^) for an accurate structure refinement from X-ray single-crystal data.

**Figure 4:**
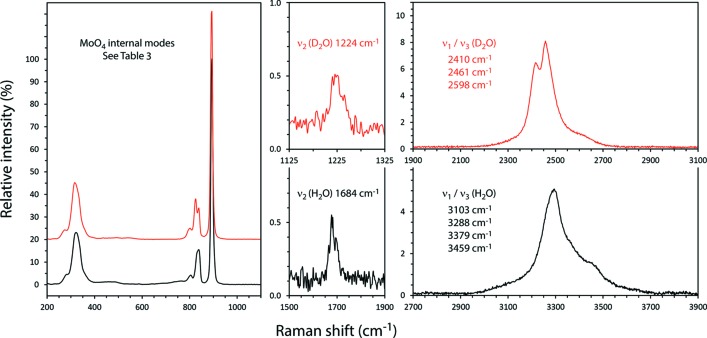
Raman spectra of Na_2_MoO_4_·2H_2_O and Na_2_MoO_4_·2D_2_O in the range 200–3900 cm^−1^. Band positions and vibrational assignments are indicated (see also Table 3 ▸). Vertical scales show intensities relative to ν_1_ (*X*O_4_^2−^).

Raman spectra of Na_2_MoO_4_·2H_2_O and Na_2_MoO_4_·2D_2_O were first reported by Mahadevan Pillai *et al.* (1997 ▸); subsequently, Luz-Lima *et al.* (2010 ▸) and Saraiva *et al.* (2013 ▸) published the Raman spectra of Na_2_MoO_4_·2H_2_O and Na_2_WO_4_·2H_2_O as a function of temperature (13–300 K) and as a function of hydro­static pressure (to 5 GPa). Both compounds exhibit evidence of a ‘conformational change’ on cooling through 120 K: the molybdate appears to undergo two high-pressure phase transitions, one at 3 GPa and the second at 4 GPa; the tungstate apparently undergoes a high-pressure phase transition at 3.9 GPa. The Raman spectra reported here (Figs. 4 ▸ and 5 ▸ and *Supporting information*) agree well with data in the literature (Table 3 ▸). The large blue-shifts in the inter­nal vibrational frequencies of the deuterated water mol­ecule are similar to the square root of the D:H mass ratio; the small blue-shifts of most of the inter­nal modes of the tetra­hedral oxyanions are consistent with stronger hydrogen bonding in the deuterated species, as expected (*cf*. Scheiner & Čuma, 1996 ▸; Soper & Benmore, 2008 ▸).

## Synthesis and crystallization   

Coarse polycrystalline powders of Na_2_MoO_4_·2H_2_O (Sigma–Aldrich M1003 > 99.5%) and Na_2_WO_4_·2H_2_O (Sigma–Aldrich 14304 > 99%) were dehydrated by drying at 673 K in air. The resulting anhydrous materials were characterised by Raman spectroscopy, X-ray and neutron powder diffraction (Fortes, 2015 ▸). This material was dissolved in D_2_O (Aldrich 151882, 99.9 atom% D) and twice recrystallized by gentle evaporation at 323 K. The molybdate crystallised with a coarse platy habit whereas the tungstate was deposited as a finer-grained material. Once the supernatant liquid was deca­nted, the residue was air dried on filter paper and then ground to a fine powder with an agate pestle and mortar. The powders were loaded into standard vanadium sample-holder tubes of inter­nal diameter 11 mm to a depth not less than 20 mm (this being the vertical neutron beam dimension at the sample position). Accurate volumes and masses were determined after the diffraction measurements were complete and used to correct the data for self-shielding. The level of deuteration was determined by Raman spectroscopy (see below) to be ∼91% for both compounds.

Raman spectra were acquired with a B&WTek *i*-Raman plus portable spectrometer; this device uses a 532 nm laser (37 mW power at the fiber-optic probe tip) to stimulate Raman scattering, which is measured in the range 170–4000 cm^−1^ with a spectral resolution of 3 cm^−1^. Data were collected for 600 sec at 17 mW for Na_2_MoO_4_·2H_2_O (as bought), 180 sec at 37 mW for Na_2_MoO_4_·2D_2_O, 300 sec at 17 mW for Na_2_WO_4_·2H_2_O (as bought) and 220 sec at 37 mW for Na_2_WO_4_·2D_2_O; after summation, the background was removed and peaks fitted using Pseudo-Voigt functions in OriginPro (OriginLab, Northampton MA). These data are provided as an electronic supplement in the form of an ASCII file. Small qu­anti­ties of ordinary hydrogen were found to be present in both specimens, the proportion being determined by the ratio of the areas under the *ν*_1_/*ν*_3_ (H_2_O) bands after normalisation relative to the height of the strong *ν*_1_ (*X*O_4_^2−^) band. The molar abundance of ^1^H was used to correct the diffraction data for absorption (see below) and to ensure accurate refinement of the structure (see *Refinement*).

**Figure 6:**
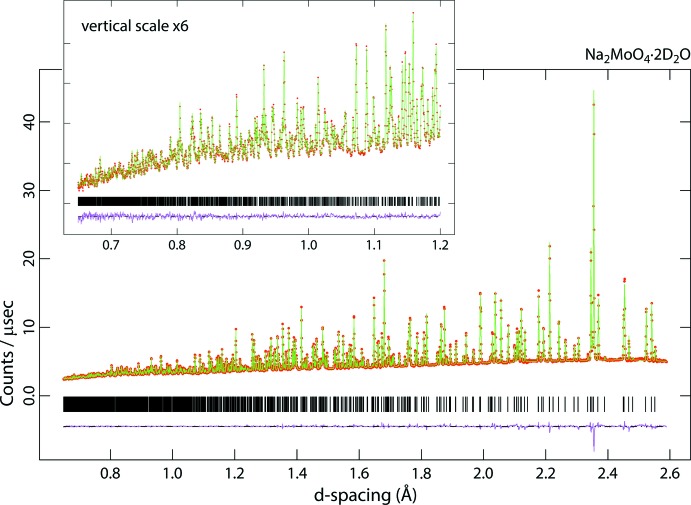
Neutron powder diffraction data for Na_2_MoO_4_·2D_2_O; red points are the observations, the green line is the calculated profile and the pink line beneath the diffraction pattern represents Obs−Calc. Vertical black tick marks report the expected positions of the Bragg peaks. The inset shows the data measured at short flight times (*i.e.* small *d*-spacings).

Time-of-flight neutron diffraction patterns were collected at 295 K using the High Resolution Powder Diffractometer, HRPD (Ibberson, 2009 ▸), at the ISIS spallation neutron source, Harwell Campus, Oxfordshire, UK. Data were acquired in the range of neutron flight times from 30–130 msec (equivalent to neutron wavelengths of 1.24–5.36 Å) for 15.17 hr from the molybdate and 14.40 hr from the tungstate, equivalent to 615 and 590 µAhr of integrated proton beam current, respectively. These data sets were normalized to the incident spectrum and corrected for detector efficiency by reference to a V:Nb null-scattering standard and then subsequently corrected for the sample-specific and wavelength-dependent self-shielding using Mantid (Arnold *et al.*, 2014 ▸: Mantid, 2013 ▸). In the case of the molybdate, the number density of the specimen was determined to be 3.28 mol nm^−3^, with a scattering cross section, allowing for the water being 9.1 mol % ^1^H, σ_scatt_ = 93.81 b and an absorption cross section, σ_abs_ = 3.66 b; for the tungstate, the number density was 3.01 mol nm^−3^, the scattering cross section, allowing for the water being 8.6 mol % ^1^H, σ_scatt_ = 94.19 b and σ_abs_ = 19.48 b. Diffraction data were exported in GSAS format and analysed with the GSAS/Expgui Rietveld package (Larsen & Von Dreele, 2000 ▸: Toby, 2001 ▸). The fitted diffraction data are shown in Figs. 6 ▸ and 7 ▸.

## Refinement   

Profile refinements were done using GSAS/Expgui (Larsen & Von Dreele, 2000 ▸; Toby, 2001 ▸) starting from the coordinates reported by Farrugia (2007 ▸). Statistically significant anisotropic displacement parameters were refined for all atoms. An assumption was made that ^1^H was uniformly distributed on all ^2^D sites, so the neutron scattering length of ^2^D was edited in GSAS in accordance with the concentration of ^1^H determined by Raman spectroscopy; for the molybdate a value of 5.776 fm was used, and for the tungstate a value of 5.724 fm was adopted. Crystal data, data collection and structure refinement details are summarized in Table 4 ▸.

## Supplementary Material

Crystal structure: contains datablock(s) Na2MoO4.2D2O, Na2WO4.2D2O, New_Global_Publ_Block. DOI: 10.1107/S2056989015011354/wm5172sup1.cif

Rietveld powder data: contains datablock(s) Na2MoO4.2D2O. DOI: 10.1107/S2056989015011354/wm5172Na2MoO4.2D2Osup2.rtv

Rietveld powder data: contains datablock(s) Na2WO4.2D2O. DOI: 10.1107/S2056989015011354/wm5172Na2WO4.2D2Osup3.rtv

CCDC references: 1406122, 1406121

Additional supporting information:  crystallographic information; 3D view; checkCIF report

## Figures and Tables

**Figure 5:**
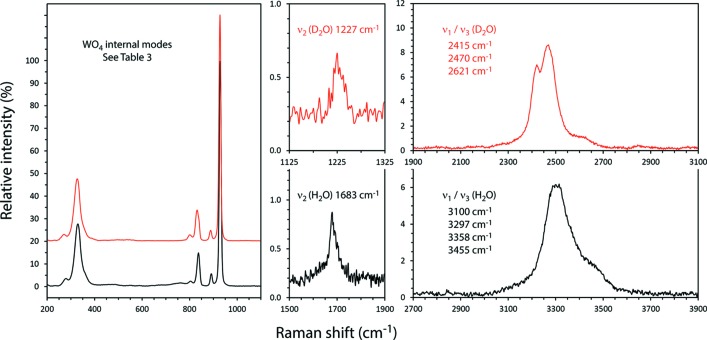
Raman spectra of Na_2_WO_4_·2H_2_O and Na_2_WO_4_·2D_2_O in the range 200–3900 cm^−1^. Band positions and vibrational assignments are indicated (see also Table 3 ▸). Vertical scales show intensities relative to ν_1_ (*X*O_4_^2−^).

**Figure 7:**
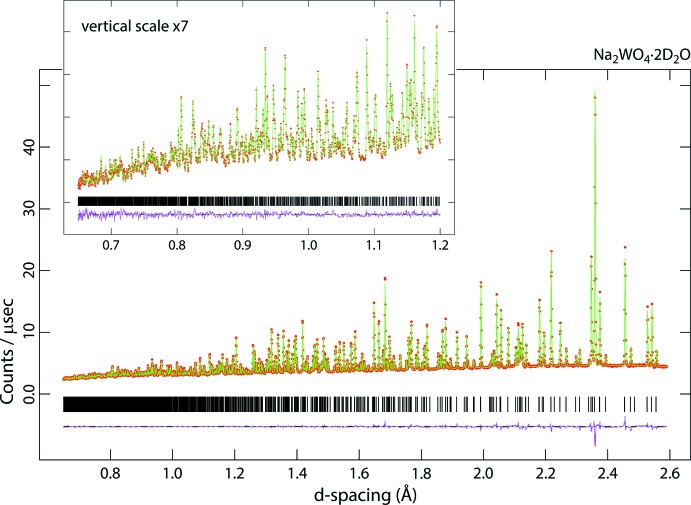
Neutron powder diffraction data for Na_2_WO_4_·2D_2_O; red points are the observations, the green line is the calculated profile and the pink line beneath the diffraction pattern represents Obs−Calc. Vertical black tick marks report the expected positions of the Bragg peaks. The inset shows the data measured at short flight times (*i.e.* small *d*-spacings).

**Table 1:** Comparison of the *X*O (*X* = Mo, W) and NaO bond lengths () in Na_2_MoO_4_2D_2_O and Na_2_WO_4_2D_2_O with those of the protonated isotopologues reported in the literature

	Na_2_MoO_4_2D_2_O	Na_2_MoO_4_2H_2_O	Na_2_WO_4_2D_2_O	Na_2_WO_4_2H_2_O
	This work	Capitelli *et al.* (2006 ▸)	This work	Farrugia (2007 ▸)
*X*O1	1.773(2)	1.772(1)	1.785(2)	1.776(3)
*X*O2	1.764(1)	1.767(1)	1.778(2)	1.778(3)
*X*O3	1.750(2)	1.751(1)	1.766(2)	1.761(3)
*X*O4	1.776(2)	1.778(1)	1.783(2)	1.787(3)
Mean *X*O	1.766	1.767	1.778	1.776
				
Na1O2	2.437(3)	2.446(2)	2.433(2)	2.442(3)
Na1O2^(i)^	2.417(3)	2.419(2)	2.412(3)	2.416(3)
Na1O3^(ii)^	2.482(3)	2.481(2)	2.479(3)	2.480(3)
Na1O4^(iii)^	2.410(3)	2.395(2)	2.399(2)	2.388(3)
Na1O5	2.476(3)	2.456(2)	2.479(3)	2.464(4)
Na1O6	2.426(3)	2.423(2)	2.443(3)	2.433(3)
Mean Na1O	2.441	2.437	2.441	2.437
				
Na2O1^iv^	2.312(3)	2.319(2)	2.320(2)	2.323(3)
Na2O2	2.363(3)	2.354(2)	2.355(2)	2.346(3)
Na2O3^v^	2.339(3)	2.341(2)	2.328(2)	2.331(3)
Na2O5	2.415(3)	2.403(2)	2.409(3)	2.396(3)
Na2O6^vi^	2.305(3)	2.300(2)	2.311(2)	2.304(3)
Mean Na2O	2.347	2.343	2.345	2.340

Symmetry codes: (i) 1*x*, 1*y*, 1*z*; (ii) 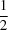
+*x*, 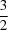 *y*, 1*z*; (iii) 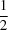 *x*, 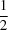
+*y*, *z*; (iv) 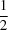
+*x*, 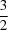 *y*, 1*z*; (v) 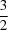 *x*, 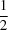
+*y*, *z*; (vi) 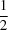
+*x*, *y*, 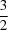 *z*.

**Table 2:** Comparison of the water molecule and hydrogen bond geometry (, ) in Na_2_MoO_4_2D_2_O and Na_2_WO_4_2D_2_O with the protonated isotopologues as reported in the literature. Note the inclusion of the contact O5D51O3, which forms the longer ‘branch’ of Farrugia’s proposed bifurcated hydrogen bond

	Na_2_MoO_4_2D_2_O	Na_2_MoO_4_2H_2_O	Na_2_WO_4_2D_2_O	Na_2_WO_4_2H_2_O
	This work	Capitelli *et al.* (2006 ▸)	This work	Farrugia (2007 ▸)
O5D51	0.977(2)	0.68(3)	0.970(2)	0.86(3)
O5D52	0.966(2)	0.76(3)	0.959(2)	0.86(3)
D51O5D52	106.0(2)	98(4)	106.0(2)	100(5)
D51O1^(i)^	1.874(2)	2.16(3)	1.873(2)	2.09(4)
O5D51O1^(i)^	167.9(2)	167(4)	168.2(2)	145(6)
D51O3^(ii)^				2.70(6)
O5D51O3^(ii)^				122(5)
D52O4^(ii)^	1.846(3)	2.07(3)	1.863(2)	1.98(3)
O5D52O4^(ii)^	171.2(2)	176(3)	170.9(2)	174(6)
				
O6D61	0.972(2)	0.83(3)	0.968(2)	0.86(3)
O6D62	0.972(2)	0.71(3)	0.966(2)	0.86(3)
D61O6D62	103.0(2)	105(3)	103.2(2)	95(5)
D61O1	1.816(2)	2.01(3)	1.834(2)	1.95(3)
O6D61O1	167.0(2)	167(3)	167.0(2)	167(6)
D62O4^(iii)^	1.868(4)	2.08(3)	1.876(2)	2.02(4)
O6D62O4^(iii)^	168.7(2)	170(3)	168.7(2)	159(6)

Symmetry codes: (i) 1*x*, 1*y*, 1*z*; (ii) 1*x*, 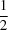
+*y*, 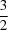 *z*; (iii) 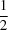
+*x*, 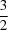 *y*, 1*z*.

**Table 3:** Comparison of the internal vibrational mode frequencies (cm^1^) in fully protonated and 90mol % deuterated isotopologues of Na_2_MoO_4_2H_2_O and Na_2_WO_4_2H_2_O with literature data

	Na_2_MoO_4_2H_2_O			Na_2_WO_4_2H_2_O		
	This work (^1^H)	This work (^2^D)	Busey Keller (1964 ▸)	This work (^1^H)	This work (^2^D)	Busey Keller (1964 ▸)
_2_ (*X*O_4_^2^)	279	271	285	276	269	276
	319	315	325	324	321	325
	335	331		330	331	
_4_ (*X*O_4_^2^)	359	358		358	355	
_3_ (*X*O_4_^2^)	804	801	805	804	802	808
	833	826	836	836	831	838
	842	840	843		840	
_1_ (*X*O_4_^2^)				891	889	893
	894	894	897	929	928	931

**Table 4:** Experimental details

	Na_2_MoO_4_2D_2_O	Na_2_WO_4_2D_2_O
Crystal data		
Chemical formula	Na_2_MoO_4_2D_2_O	Na_2_WO_4_2D_2_O
*M*_r_	245.99	333.87
Crystal system, space group	Orthorhombic, *P* *b* *c* *a*	Orthorhombic, *P* *b* *c* *a*
Temperature (K)	295	295
*a*, *b*, *c* ()	8.482961(14), 10.566170(17), 13.83195(3)	8.482514(15), 10.595156(19), 13.85640(3)
*V* (^3^)	1239.79(1)	1245.32(1)
*Z*	8	8
Radiation type	Neutron	Neutron
(mm^1^)	0.03 + 0.0007 *	0.03 + 0.0033 *
Specimen shape, size (mm)	Cylinder, 38 11	Cylinder, 50 11

Data collection		
Diffractometer	HRPD, High resolution neutron powder	HRPD, High resolution neutron powder
Specimen mounting	Vanadium tube	Vanadium tube
Data collection mode	Transmission	Transmission
Scan method	Time of flight	Time of flight
Absorption correction	Analytical [data were corrected for self shielding using _scatt_ = 93.812 barns and _ab_() = 3.657 barns at 1.798 during the normalization procedure. The linear absorption coefficient is wavelength dependent and is calculated as: = 0.0308 + 0.0007 * (mm^1^)]	analytical [data were corrected for self shielding using _scatt_ = 94.190 barns and _ab_() = 19.484 barns at 1.798 during the normalization procedure. The linear absorption coefficient is wavelength dependent and is calculated as: = 0.0284 + 0.0033 * (mm^1^)]
*T*_min_, *T*_max_	0.685, 0.706	0.700, 0.603
2 values ()	2_fixed_ = 168.329	2_fixed_ = 168.329
Distance from source to specimen (mm)	95000	95000
Distance from specimen to detector (mm)	965	965

Refinement		
*R* factors and goodness of fit	*R*_p_ = 0.013, *R*_wp_ = 0.013, *R*_exp_ = 0.007, *R*(*F* ^2^) = 0.05255, ^2^ = 3.534	*R*_p_ = 0.014, *R*_wp_ = 0.013, *R*_exp_ = 0.007, *R*(*F* ^2^) = 0.04597, ^2^ = 3.312
No. of data points	4610	4610
No. of parameters	133	133

Computer programs: HRPD control software, *GSAS*/Expgui (Larsen Von Dreele, 2000 ▸: Toby, 2001 ▸), Mantid (Arnold *et al.*, 2014 ▸: Mantid, 2013 ▸), *DIAMOND* (Putz Brandenburg, 2006 ▸) and *publCIF* (Westrip, 2010 ▸).

## supplementary crystallographic information

### Crystal data

**Table:**

Na_2_WO_4_·2D_2_O	*D*_x_ = 3.562 Mg m^−^^3^
*M**_r_* = 333.87	Melting point: 373 K
Orthorhombic, *P**b**c**a*	Neutron radiation
Hall symbol: -P 2ac 2ab	µ = 0.03+ 0.0033 * λ mm^−^^1^
*a* = 8.482514 (15) Å	*T* = 295 K
*b* = 10.595156 (19) Å	white
*c* = 13.85640 (3) Å	cylinder, 50 × 11 mm
*V* = 1245.32 (1) Å^3^	Specimen preparation: Prepared at 323 K and 100 kPa
*Z* = 8	

### Data collection

**Table:**

HRPD, High resolution neutron powder diffractometer	Absorption correction: analytical Data were corrected for self shielding using σ_scatt_ = 94.190 barns and σ_ab_(λ) = 19.484 barns at 1.798 Å during the normalisation procedure. The linear absorption coefficient is wavelength dependent and is calculated as: µ = 0.0284 + 0.0033 * λ [mm^-1^]
Radiation source: ISIS Facility, Neutron spallation source	*T*_min_ = 0.603, *T*_max_ = 0.700
Specimen mounting: vanadium tube	2θ_fixed_ = 168.329
Data collection mode: transmission	Distance from source to specimen: 95000 mm
Scan method: time of flight	Distance from specimen to detector: 965 mm

### Refinement

**Table:**

Least-squares matrix: full	Excluded region(s): none
*R*_p_ = 0.014	Profile function: TOF profile function #3 (21 terms). Profile coefficients for exp pseudovoigt convolution [Von Dreele, 1990 (unpublished)] (α) = 0.1414, (β_0_) = 0.026250, (β_1_) = 0.004690, (σ_0_) = 0, (σ_1_) = 322.9, (σ_2_) = 15.7, (γ_0_) = 0, (γ_1_) = 0, (γ_2_) = 0, (γ_2s_) = 0, (γ_1e_) = 0, (γ_2e_) = 0, (ε_i_) = 0, (ε_a_) = 0, (ε_A_) = 0, (γ_11_) = 0.023, (γ_22_) = 0, (γ_33_) = 0.006, (γ_12_) = 0.050, (γ_13_) = 0.016, (γ_23_) = 0.017. Peak tails ignored where intensity <0.0010x peak. Aniso. broadening axis 0.0 0.0 1.0
*R*_wp_ = 0.013	133 parameters
*R*_exp_ = 0.007	0 restraints
*R*(*F*^2^) = 0.04597	0 constraints
χ^2^ = 3.312	(Δ/σ)_max_ = 0.04
4610 data points	Background function: GSAS Background function number 1 with 12 terms. Shifted Chebyshev function of 1st kind 1: 3.91163, 2: 1.22805, 3: -0.206144, 4: -8.53351x10^-2^, 5: -9.966470x10^-2^, 6: -1.847470x10^-2^, 7: -1.38195x10^-2^, 8: 9.956170x10^-4^, 9: 4.49839x10^-3^, 10: -2.199010x10^-2^, 11: 2.57524x10^-2^, 12: -2.00574x10^-3^

### Fractional atomic coordinates and isotropic or equivalent isotropic displacement parameters (Å^2^)

**Table:**

	*x*	*y*	*z*	*U*_iso_*/*U*_eq_	
W1	0.51352 (13)	0.80186 (10)	0.52310 (10)	0.01206	
Na1	0.3444 (2)	0.4957 (2)	0.58501 (16)	0.02213	
Na2	0.7422 (2)	0.54966 (18)	0.64745 (14)	0.02166	
O1	0.44940 (14)	0.82253 (11)	0.40144 (8)	0.01858	
O2	0.55647 (14)	0.63936 (9)	0.54135 (9)	0.01675	
O3	0.68666 (14)	0.89213 (10)	0.53870 (10)	0.02256	
O4	0.36916 (14)	0.85058 (11)	0.60895 (9)	0.01972	
O5	0.53794 (17)	0.40814 (14)	0.70116 (12)	0.02505	
O6	0.2276 (2)	0.64134 (14)	0.70148 (11)	0.02531	
D51	0.5576 (2)	0.32926 (17)	0.66767 (12)	0.03829	
D52	0.55912 (18)	0.39189 (14)	0.76800 (13)	0.03325	
D61	0.1229 (2)	0.64645 (13)	0.67384 (10)	0.03267	
D62	0.27774 (17)	0.71764 (15)	0.67874 (12)	0.03524	

### Atomic displacement parameters (Å^2^)

**Table:**

	*U*^11^	*U*^22^	*U*^33^	*U*^12^	*U*^13^	*U*^23^
W1	0.0099 (7)	0.0064 (6)	0.0199 (7)	−0.0002 (5)	0.0009 (6)	0.0002 (6)
Na1	0.0209 (11)	0.0167 (9)	0.0289 (13	0.0004 (9)	0.0028 (9)	0.0003 (8)
Na2	0.0192 (10)	0.0183 (11)	0.0275 (13)	0.0001 (8)	−0.0006 (8)	0.0000 (9)
O1	0.0170 (6)	0.0192 (6)	0.0195 (7)	0.0008 (5)	0.0010 (6)	0.0031 (5)
O2	0.0176 (6)	0.0079 (5)	0.0248 (7)	0.0014 (5)	−0.0004 (5)	0.0035 (5)
O3	0.0193 (7)	0.0190 (6)	0.0293 (8)	−0.0083 (5)	−0.0002 (6)	−0.0015 (6)
O4	0.0201 (6)	0.0176 (6)	0.0214 (7)	0.0045 (5)	0.0052 (6)	0.0008 (6)
O5	0.0305 (9)	0.0206 (9)	0.0241 (8)	−0.0027 (7)	−0.0031 (7)	−0.0023 (7)
O6	0.0246 (8)	0.0247 (8)	0.0266 (9)	−0.0004 (7)	−0.0046 (7)	0.0061 (7)
D51	0.0448 (10)	0.0304 (9)	0.0397 (9)	−0.0001 (8)	−0.0104 (8)	−0.0032 (8)
D52	0.0415 (9)	0.0323 (8)	0.0259 (8)	−0.0027 (7)	−0.0001 (8)	0.0004 (7)
D61	0.0259 (9)	0.0354 (9)	0.0367 (9)	−0.0011 (7)	−0.0030 (7)	0.0046 (8)
D62	0.0347 (9)	0.0270 (8)	0.0440 (11)	−0.0059 (7)	−0.0029 (7)	0.0059 (7)

### Geometric parameters (Å, º)

**Table:**

W1—O1	1.7849 (19)	Na2—O3^v^	2.328 (2)
W1—O2	1.7779 (15)	Na2—O5	2.409 (3)
W1—O3	1.7659 (17)	Na2—O6^vi^	2.311 (2)
W1—O4	1.7834 (18)	O5—D51	0.9702 (18)
Na1—O2	2.433 (2)	O5—D52	0.9591 (16)
Na1—O2^i^	2.412 (3)	O6—D61	0.9684 (16)
Na1—O3^ii^	2.479 (3)	O6—D62	0.9664 (16)
Na1—O4^iii^	2.399 (2)	D51—O1^i^	1.873 (2)
Na1—O5	2.479 (3)	D52—O4^vii^	1.863 (2)
Na1—O6	2.443 (3)	D61—O1^ii^	1.834 (2)
Na2—O1^iv^	2.320 (2)	D62—O4	1.876 (2)
Na2—O2	2.355 (2)		
			
O1—W1—O2	108.40 (9)	O2^i^—Na1—O6	174.55 (12)
O1—W1—O3	107.61 (8)	O4^iii^—Na1—O5	99.82 (9)
O1—W1—O4	112.66 (8)	O4^iii^—Na1—O6	90.40 (8)
O2—W1—O3	109.67 (7)	O5—Na1—O6	94.37 (10)
O2—W1—O4	109.03 (9)	O1^iv^—Na2—O2	95.10 (8)
O3—W1—O4	109.43 (9)	O1^iv^—Na2—O3^v^	91.90 (8)
O2—Na1—O2^i^	86.16 (7)	O1^iv^—Na2—O5	176.73 (11)
O2—Na1—O3^ii^	85.82 (8)	O1^iv^—Na2—O6^vi^	93.43 (9)
O2—Na1—O4^iii^	173.55 (11)	O2—Na2—O3^v^	93.36 (8)
O2—Na1—O5	84.60 (8)	O2—Na2—O5	87.87 (7)
O2—Na1—O6	93.95 (9)	O2—Na2—O6^vi^	111.09 (9)
O3^ii^—Na1—O2^i^	88.32 (8)	O3^v^—Na2—O5	86.57 (8)
O3^ii^—Na1—O4^iii^	89.72 (8)	O3^v^—Na2—O6^vi^	154.35 (11)
O3^ii^—Na1—O5	170.42 (10)	O5—Na2—O6^vi^	86.74 (9)
O3^ii^—Na1—O6	86.26 (8)	D51—O5—D52	105.96 (19)
O2^i^—Na1—O4^iii^	89.05 (8)	D61—O6—D62	103.18 (19)
O2^i^—Na1—O5	91.07 (9)		

Symmetry codes: (i) −*x*+1, −*y*+1, −*z*+1; (ii) *x*−1/2, −*y*+3/2, −*z*+1; (iii) −*x*+1/2, *y*−1/2, *z*; (iv) *x*+1/2, −*y*+3/2, −*z*+1; (v) −*x*+3/2, *y*−1/2, *z*; (vi) *x*+1/2, *y*, −*z*+3/2; (vii) −*x*+1, *y*−1/2, −*z*+3/2.
